# The Extents of Coronary Heart Disease and the Severity of Newly Developed Dry Eye Disease: A Nationwide Cohort Study

**DOI:** 10.3390/diagnostics14060586

**Published:** 2024-03-10

**Authors:** Chia-Yi Lee, Shun-Fa Yang, Jing-Yang Huang, Chao-Kai Chang

**Affiliations:** 1 Institute of Medicine, Chung Shan Medical University, Taichung 402, Taiwan; 2 Nobel Eye Institute, Taipei 115, Taiwan; 3 Department of Ophthalmology, Jen-Ai Hospital Dali Branch, Taichung 412, Taiwan; 4 Department of Medical Research, Chung Shan Medical University Hospital, Taichung 402, Taiwan; 5 Department of Optometry, Da-Yeh University, Chunghua 515, Taiwan

**Keywords:** coronary heart disease, dry eye disease, age, severity, epidemiology

## Abstract

This study aimed to evaluate the potential association between coronary heart disease (CHD) severity and the subsequent dry eye disease (DED) with a different severity through the use of the National Health Insurance Research Database (NHIRD) of Taiwan. A retrospective cohort study was conducted. The CHD population was further divided into a severe CHD that had received coronary artery bypass graft (CABG) surgery group and a mild CHD that had received medicine group, then matched with a 1:2 ratio, and 29,852 and 14,926 CHD patients were put into the severe CHD and mild CHD groups, respectively. The primary outcomes were the development of DED and severe DED after CHD diagnosis. The Cox proportional hazards regression was used to produce the adjusted hazard ratio (aHR) and 95% confidence interval (CI) of DED and severe DED between groups. There were 3440 and 1276 DED cases in the mild CHD and severe CHD groups, respectively. And another 37 and 48 severe CHD events were observed in the mild and severe CHD groups, respectively. The incidence of severe DED in the severe CHD group was significantly higher compared to the mild CHD group (aHR: 5.454, 95% CI: 1.551–7.180, *p* = 0.0001). The cumulative probabilities of DED and severe DED were significantly higher in the severe CHD group than the mild CHD group (both *p* < 0.0001). In the subgroup analysis, the correlation between severe CHD and DED was higher in the patients aged older than 70 years (*p* < 0.0001). In conclusion, severe CHD is associated with a higher incidence of severe DED with a higher cumulative incidence.

## 1. Introduction

Coronary heart disease (CHD) is a major disease of the human population that presents with coronary artery stenosis and myocardial ischemia [1], and the prevalence of CHD can elevate to above 27 percent in the Asian population [2]. Mild CHD is a relatively chronic disease that can be managed using medical treatments like anti-platelet medications and anti-lipid medications [3,4]. In contrast, severe CHD would cause significant coronary artery occlusion and surgical management is usually warranted to prevent the mortality [5]. A coronary artery bypass graft (CABG) surgery can be applied to replace the obstructed coronary vessel and recover cardiac circulation [6,7].

In addition to cardiac tissue, the existence of CHD can affect and deteriorate the condition of other organs [8,9,10]. Behcet’s disease is an inflammatory disease that contributes to systemic inflammation and other coronary arterial damage [11,12]. Also, individuals with CHD are at a higher risk of developing several metabolic syndromes like hypertension and diabetes mellitus (DM) [13,14]. Moreover, periodontitis development was associated with the degree of CHD in a previous publication [15]. For the influence of CHD on an ophthalmic disorder, a higher risk of open angle glaucoma development was observed in individuals diagnosed with CHD [16].

Dry eye disease (DED) is a prevalent inflammatory eye disease and the incidence of DED reached 60 percent in the Japanese population of visual display terminal users [17]. Regarding the type of DED, evaporative DED presents with the excessive evaporation of the tear film from the ocular surface, which is related to meibomian gland dysfunction (MGD) and tear film instability [18,19,20]. Another common type of DED is aqueous-deficient DED, which features a lacrimal deficiency and a subsequently reduced tear menisci height and radius of curvature [19], and the previous literature has demonstrated the significant correlation between aqueous-deficient DED and inflammatory syndromes like Sjogren syndrome, rheumatic arthritis, and diabetes mellitus (DM) [21]. Concerning the relationship between DED and CHD, a previous study demonstrated the increasing risk of a cardiovascular event in patients with primary Sjogren syndrome [22]. Still, whether the severity of CHD would affect the incidence of DED remains unknown. Since an inflammatory reaction would elevate in both CHD and DED [1,19], a prominent association between CHD severity and the DED rate may be possible.

Consequently, the purpose of the current study is to investigate the potential correlation between the severity of CHD and the following DED through the usage of the National Health Insurance Research Database (NHIRD) in Taiwan. The associations of CHD severity with different severities of DED were also evaluated in the current study.

## 2. Materials and Methods

### 2.1. Data Source

The NHIRD of Taiwan has the medical documents of 23 million Taiwanese persons from the 1 January 2000 to the 31 December 2020. The feasible medical records in the NHIRD include the International Classification of Diseases Ninth Revision (ICD-9) diagnostic code, the International Classification of Diseases Tenth Revision (ICD-10) diagnostic code, age, sex, employment, income degree, educational degree, urbanization degree, image codes, laboratory codes, the medical department codes, the procedure codes, surgical codes, and the international ATC codes for medications which were offered by the national health insurance system.

### 2.2. Patient Inclusion

**Figure 1:**
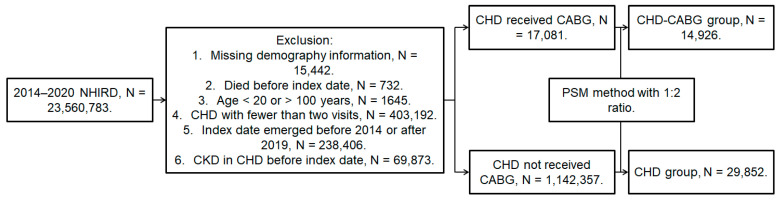
The flowchart of subject selection. CHD: coronary heart disease, CABG: coronary artery bypass graft, DED: dry eye disease, N: number, NHIRD: National Health Insurance Research Database.

A retrospective cohort study was attended and individuals were defined as bearing CHD if they fulfilled these conditions: (1) the receipt of a CHD diagnoses according to the ICD-9 or ICD-10 codes from 2014 to 2019; (2) adequate examinations including their complete blood cell count, white blood cell differentiation count, triglyceride, cholesterol, low-density lipoprotein, high-density lipoprotein, an electrocardiogram, and a cardiac angiography that were done before CHD diagnosis; (3) aged from 20 to 100 years old; and (4) patients had followed up in general internal medicine, family medicine, or the cardiovascular department for at least three months. Only data of the NHIRD during 2014–2019 rather than 2000–2020 were used because the authors wanted to demonstrate the trend in the data within ten years from now to enhance the timeliness of the data. These things considered, a follow-up period for at least one year should be achieved; thus, those patients that emerged in 2020 (the end year of the NHIRD) were excluded. The index date of the current study was 6 months after initial CHD diagnosis. Moreover, patients would be excluded if they met these criteria: (1) no demographic data, (2) death before the index date, (3) an index date after 2019 or before 2014, and (4) DED that developed before the index date. To survey the effect of CHD severity, all CHD patients were segregated into the mild CHD group which received medications and the severe CHD group which received CABG surgery. One severe CHD patient who had undergone CABG surgery was matched with two mild CHD patients on medications by the propensity score-matching (PSM) approach that matched the demographic data, co-morbidities, and medications between severe the CHD patients who received CABG surgery and the mild CHD patients who received medicines. After the PSM approach, a total of 29,852 and 14,926 CHD patients were put into the severe CHD and mild CHD groups, respectively. The flowchart of patient inclusion is illustrated in Figure 1.

### 2.3. Primary Outcome

The primary outcome in the current study was regarded to be the DED and severe DED developments. Patients were defined as having DED if they reached these criteria: (1) the receipt of ICD-9 and ICD-10 diagnostic codes for DED, (2) the arrangement of a slit-lamp biomicroscope exam, tear break-up time or Schirmer test before the DED diagnosis via exam codes, and (3) the DED diagnosis was done by an ophthalmologist. Generally, the ophthalmologists in Taiwan diagnosed DED if the DED-related symptoms were presented in addition to a tear break-up time lower than 10 s and a Schirmer test lower than 10 mm, which were similar to previous criteria [23]. Furthermore, severe DED was defined as (1) meeting the criteria of simple DED and (2) receiving a prescription for a topical cyclosporine eyedrop via ATC codes. Except the ophthalmologists in Taiwan who used the criteria similar to DEWS for severe DED diagnosis [23], the application of cyclosporine A for severe DED needed to be approved by the National Health Insurance Administration after evaluating the tear secretion status via a Schirmer test and corneal surface image with a fluorescein stain. Consequently, the credibility of cyclosporine A usage as a criteria of severe DED might be adequate. Only the DED or severe DED events that occurred after the index date were seen as the primary outcome in the current study. The CHD patients in the current study were followed until DED/severe DED development, they were withdrawn from the National Health Insurance program, or the end of the NHIRD indicated 31 December 2020.

### 2.4. Related Covariates

Some demographic data, co-morbidities, and medications were enrolled in the multivariable analysis to adjust the influence of related covariates for DED development: sex, age, occupation, hypertension, DM, hyperlipidemia, cerebrovascular disease, rheumatoid arthritis, systemic lupus erythematosus, Sjogren syndrome, the receipt of cataract surgery, an antihistamine, biguanides, a dipeptidyl peptidase-4 inhibitor, sodium-glucose cotransporter-2 inhibitors, beta-blockers, statin, diuretic and benzodiazepine. The inclusion of potential covariates was in accordance with previous experience [10,22,24,25], while the enrollment of vascular disorders and anti-diabetic medications could standardize the general health status and present the severity of DM, respectively. The definitions of these covariates were defined by means of the demographic codes, the ICD-9 and ICD-10 diagnostic codes, and the ATC codes in the Taiwan NHIRD. To confirm that these covariates persisted long enough to alter the development of DED, only the covariates that had been recorded in the NHIRD for more than two years before the index date were deemed relevant to the current study. 

### 2.5. Statistical Analysis

SAS version 9.4 (SAS Institute Inc., Cary, NC, USA) was used for statistical analyses in the current study. The descriptive analysis and absolute standardized difference (ASD) were applied to evaluate the distributions of clinical characters between the two groups, and an ASD value higher than 0.1 was defined as a significant difference in the current study, according to previous experience [26]. Then, the Cox proportional hazard regression was administered to calculate the adjusted hazard ratios (aHRs) with 95% confidence intervals (CI) of DED and severe DED formations between the severe CHD group and mild CHD group. The hazard ratio referred to the relationship between the instantaneous hazards in the two groups and the aHR mean of the same relationship but modified by several covariates. The multivariable Cox proportional hazard regression, which measures the risk of failure (i.e., outcome achievement) with the consideration of survival time, was used for the production of an aHR between the severe CHD and mild CHD groups. In addition, the null hypothesis is that the incidence of DED/severe DED in the severe CHD population is similar to the incidence of DED/severe DED in the mild CHD population. The multivariable Cox proportional hazard regression of the SAS version 9.4 (SAS Institute Inc., Cary, NC, USA) software was used and the possible effect of demographic data, co-morbidities including DM, and medicines including anti-hyperglycemic medications on DED development were adjusted in the multivariable analysis/Cox proportional hazard regression to control for the covariates of DED formation as much as possible. This method/model included multiple covariates, like demographic data, co-morbidities, and co-medications, and removed their effect while analyzing the correlation between CHD and DED. The Kaplen–Meier curve was plotted to illustrate the cumulative incidence of DED and severe DED between the mild CHD and severe CHD groups and a log-rank test was used to analyze the cumulative incidence between the two groups. In subgroup analyses, CHD patients were classified by both age and sex, and the Cox proportional hazard regression was executed again to evaluate the aHR and 95% CI of the DED and severe DED of individuals with severe CHD compared to individuals with mild CHD in each subgroup. The interaction test was utilized to compare the different influence of CHD severity on DED severity based on age and sex stratifications. The statistical significance was set as *p* < 0.05 in the current study.

## 3. Results

The clinical characteristics of the mild CHD and severe CHD populations are shown in Table 1. The ratios of sex were identical between the two groups and the distributions of age also showed no difference between the two groups (both ASD < 0.1000). The occupation types and all the co-morbidities except DM (ASD = 0.1945) demonstrated similar distributions between the two groups (all ASD < 0.1000). Regarding the medication prescriptions, the severe CHD group showed a higher ratio of dipeptidyl peptidase-4 inhibitor application compared to the mild CHD group (ASD = 0.3872), while the applications of other medications did not show a significant difference between the two groups due to the PSM approach (all ASD < 0.1000) (Table 1).

**Figure 2:**
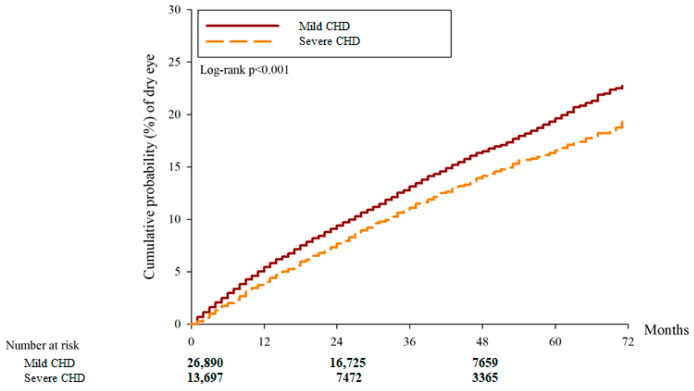
The cumulative incidence of dry eye disease between the two groups. CHD: coronary heart disease.

**Figure 3:**
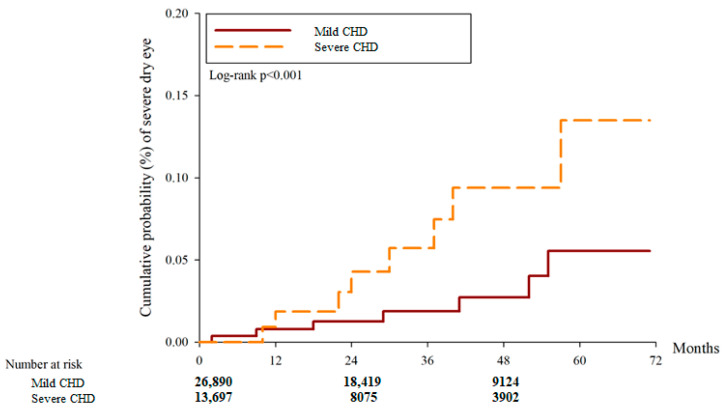
The cumulative incidence of severe dry eye disease between the two groups. CHD: coronary heart disease.

After the study interval, there were 3440 and 1276 DED cases in the mild CHD and severe CHD groups, respectively. And another 37 and 48 severe DED events were observed in the mild and severe CHD groups, respectively. According to the multivariable analysis, the incidence of DED between the two groups was similar (aHR: 0.847, 95% CI: 0.784–1.016, *p* = 0.8213) while the severe CHD group showed a higher incidence of severe DED compared to the mild CHD group (aHR: 5.454, 95% CI: 1.551–7.180, *p* = 0.0001) (Table 2). Concerning cumulative probability, both the cumulative probabilities of DED and severe DED were significantly higher in the severe CHD group than the mild CHD group (both *p* < 0.0001) (Figure 2 and Figure 3). The correlations between DED development and other potential risk factors are presented in Table S1.

In the subgroup analysis, the correlations of CHD severity and DED development were similar between the different sex subgroups (*p* = 0.5700). Also, the correlations of CHD severity and severe DED did not demonstrate a significant difference between the male and female populations (*p* = 0.0584) (Table 3). For age, the correlation between severe CHD and DED development was higher in patients aged older than 70 years compared to their younger counterparts (*p* < 0.0001). On the other hand, the correlation between severe CHD and a following severe DED development did not illustrate a significant difference among the different age subgroups (*p* = 0.6112) (Table 4).

## 4. Discussion

In the current study, the patients with severe CHD were at a higher risk of developing severe DED compared to the patients with mild CHD. Also, the cumulative probability of both DED and severe DED were significantly higher for the patients with severe CHD compared to the mild CHD cases. On the other hand, the correlation between severe CHD and DED increased in patients aged older than 70 years old.

The development of CHD can damage the heart, coronary artery, and other organs, as stated in the previous literature, due to several mechanisms [9,27,28]. The inflammatory reaction is a major pathway that leads to the formation and development of CHD, in which the neutrophil-to-lymphocyte ratio (an inflammatory marker) correlates with CHD formation and can serve as a significant predictor of CHD’s development [29]. In addition, the levels of plasma cytokines like interleukin and C-reactive protein were higher in patients who were diagnosed with CHD [30]. In addition, certain systemic inflammatory diseases such as inflammatory bowel disease and ankylosing spondylitis are associated with the formation of CHD [31,32]. In addition to inflammation, hyperlipidemia is another mechanism of CHD which can contribute to the formation of atherosclerotic plaque as well as coronary arterial stenosis [33]. Atherosclerotic plaque and the following CHD could be caused by the higher expression of triglyceride [34], and a higher LDL level is an established predisposing factor for the formation of CHD [34]. In addition, oxidative stress also plays a crucial role in the development of CHD which significantly associates with the formation of acute cardiovascular morbidities [35]. DED is an ocular inflammatory disease that features tear film instability and increments of cytokines [36]. In a previous study, both the expressions of interleukin-6 and matrix metalloproteinases were significantly higher in patients with DED [19]. In addition, several systemic diseases including Sjogren syndrome and systemic lupus erythematous serve as predisposing factors for DED development [21,37]. On the other side, the oxidative stress on the ocular surface is also elevated in patients with prominent DED [19]. Regarding the association between hyperlipidemia and DED development, a previous study presented the higher ratio of hyperlipidemia and statin use in the DED population [38]. Because CHD and DED share a similar pathophysiology [19,30], severe CHD may indicate an activation of those mechanisms and the incidence of DED may be higher in such a circumstance. This concept was supported by the findings of the current study.

In the current study, severe CHD was associated with a higher incidence of a following severe DED that needed cyclosporine to manage it. In previous studies, a higher ratio of CHD has been reported in the DED population [10,22]. Still, whether this relationship will persist inversely was not elucidated and the average patient numbers in those studies were low [10,22]. The current study may be a preliminary experience to demonstrate the correlation between CHD severity and the incidence of subsequent severe DED in an adequate study population. DED episodes before the index date, which was indicated to be six months after CHD diagnosis, were excluded; thus, pre-existing DED would not interfere with the statistical analysis of the current study. Moreover, the effect of several risk factors of DED were considered in the Cox proportional hazard regression, which included age, sex, DM, rheumatic arthritis, systemic lupus erythematous, Sjogren syndrome, cataract surgery, and certain medications [10,22,24]. Because the two groups were matched according to the score that consisted of all the diseases mentioned above, some diseases may show a higher trend in one group. The more severe DM in the severe CHD group may result from the significant correlation between CHD and DM that has been reported in the previous literature [14]; thus, the usage of DPP-4 was more frequent in such a population because DPP-4 is the second line DM medications (for moderate to severe DM) in Taiwan. Furthermore, the DM-related factors were controlled by adjusting for the effect of DM and anti-hyperglycemic medications on the development of DED in the Cox proportional hazard regression. The results showed that severe CHD is still a significant risk factor for severe DED after controlling for the effect of multiple risk factors including DM and anti-hyperglycemic medications. As a consequence, severe CHD could be an independent risk factor for the development of subsequent severe DED. The inflammatory reaction in the severe DED population is usually higher than that in the mild DED population [19]; thus, several immunosuppressants including cyclosporine and tacrolimus have been applied to manage severe DED [20,36]. Accordingly, severe CHD with a higher inflammatory response may trigger general-to-severe DED more easily. The cumulative probability of severe DED also indicates the incidence of severe DED associated with a prolonged CHD course and probably persistent inflammation. On the other hand, the incidence of DED did not show a significant difference between the severe CHD and mild CHD groups, while the cumulative probability of DED was significantly higher in the severe CHD population than the mild CHD population. A possible explanation is that the accumulation of DED events is faster in the severe CHD group despite the overall similar incidence, and the DED incidence in the severe CHD group was also marginally higher than that in the mild CHD group at the end of the follow-up period. A possible explanation is that the overall incidence of DED might be significantly higher in the severe CHD population than in the mild CHD population if a longer follow-up period were to be arranged. Still, further research is warranted to prove this concept.

In the subgroup analysis, the associations between severe CHD and a following DED were similar between the male and female populations according to the interaction test. However, the aHR of DED in the female population with severe CHD was numerically higher than that in the male population with severe CHD. Furthermore, the aHR of severe DED in women with severe CHD was also numerically higher than the aHR in the male population with severe CHD despite the insignificant difference. The female sex is a known risk factor for the development of DED, with about a 2.68 folds–hazard ratio [39]. The results of the sex-based subgroup analysis in the current study may have implied that females still are at a higher risk of DED development compared to the male population, which corresponded with the results of a previous publication [39]. Concerning the age-based subgroup analysis, the correlation between severe CHD and DED was more prominent in the severe CHD patients that were aged older than 70 years, with significance. There was a rare study to present this phenomenon. The possible explanation for this phenomenon is that old age is a well-known risk factor for the development of general DED [39]; thus, the effect of old age persisted in the specific population with severe CHD. However, the correlation between severe CHD and severe DED became insignificant among the different age subgroups. There has been little research to discuss the potential risk factors for severe DED. A possible reason for the insignificant effect of old age on severe DED development might be due to the chronic inflammatory reaction in the elderly compared to the younger group [40]; thus, the effect of inflammation from severe CHD on severe DED was diluted. Also, the elderly could be suffering from more systemic diseases which the ophthalmic consultant may not give a high priority to, and thus, some severe DED in this population could be omitted.

When it comes to epidemiology, CHD is a common vascular disease that above five percent of the Caucasian population has been diagnosed with [41,42]. Severe CHD, which indicates the presence of advanced coronary atherosclerosis, was found in 30 percent of the whole CHD population, although the incidence has decreased recently [43]. And the presence of CHD can cause a considerable mortality rate that has achieved 40 percent in the European male population with an adequate follow-up period [44]. DED, similar to CHD, affects numerous individuals throughout the world with an annual incidence of above six percent in individuals older than 40 years old [21]. Severe DED can not only affect the ocular surface but can cause prominent visual loss and psychological stress [21]. Since both CHD and DED influence a majority of the population and could result in a huge socioeconomic burden, any correlation between them could be evaluated and illustrated.

There are several limitations in the current study. Due to the claimed database application, some important information like the image results of CHD, laboratory results of CHD, the surgical details of the CABG surgery, the postoperative condition of the CABG surgery, the long-term blood pressure and blood lipid levels of CHD, the recurrence of major coronary obstruction in CHD patients, the severity of co-morbidities, the results of tear break-up time as well as the Schirmer test in DED patients, the image results like the external eye photography of DED patients, the dose of cyclosporine in each DED patient, and the treatment outcome of the DED population including the improvement of signs and symptoms cannot be accessed and the integrity of the results of this research could be reduced. Although the diagnosis of DED in Taiwan is generally similar to the DEWS criteria [23], the absence of real medical records in the NHIRD would diminish the accuracy of DED diagnosis. The usage of cyclosporine A plus a DED diagnosis was regarded as the criteria of severe DED in the current study, but not all the severe DED cases applied cyclosporine A in Taiwan and the results of DED exams were also absent; thus, the number of severe DED cases may be underestimated. Additionally, CABG surgery was used as the severity index of CHD while the standard of arranging CABG surgery may be different for each cardiac specialist. Similarly, the application of cyclosporine A was used to defined the presence of severe DED, but the usage of cyclosporine may be subjective. Also, some patients that visited a local clinic may receive self-paid cyclosporine treatment; thus, the incidence of severe DED may be underestimated under such criteria. Finally, the total number of severe DED cases was relatively low, in which only 85 cases were recorded during the whole study interval, and the low outcome numbers may lead to statistical bias.

## 5. Conclusions

In conclusion, the presence of severe CHD is correlated with a higher incidence of subsequent severe DED after adjusting for multiple covariates. Furthermore, the cumulative incidences of both DED and severe DED increases in patients with severe CHD. Consequently, a more aggressive DED treatment may be suggested to patients with severe CHD. Further prospective large-scale studies to survey the correlation between CHD severity and different types of DED, and the influence of CHD severity on the treatment outcome of DED are mandatory.

## Figures and Tables

**Table 1:** The characteristics between the mild and severe coronary heart disease populations.

Character	Mild CHD Group (*N* = 29,852)	Severe CHD Group (*N* = 14,926)	ASD
Sex			<0.0001
Male	22,132 (74.14%)	11,066 (74.14%)	
Female	7720 (25.86%)	3860 (25.86%)	
Age			0.0009
<40	472 (1.58%)	223 (1.48%)	
40–49	1987 (6.67%)	1014 (6.79%)	
50–59	6020 (20.16%)	2967 (19.88%)	
60–69	10,775 (36.09%)	5465 (36.60%)	
>=70	10,598 (35.50%)	5257 (35.22%)	
Occupation			0.0016
Government employee	1488 (4.98%)	610 (4.09%)	
Worker	15,348 (51.41%)	7899 (52.92%)	
Farmer and fisherman	5876 (19.68%)	2377 (15.93%)	
Low-income	442 (1.48%)	231 (1.55%)	
Others	6698 (22.44%)	3809 (25.52%)	
Co-morbidity			
Hypertension	19,470 (65.23%)	12,207 (81.79%)	0.0852
DM	9192 (30.78%)	8711 (58.36%)	0.1945 *
Hyperlipidemia	13,023 (43.63%)	9048 (60.62%)	0.0695
Cerebrovascular disease	2917 (9.77%)	2315 (15.51%)	0.0303
Rheumatoid arthritis	293 (0.97%)	138 (0.91%)	0.0004
Systemic lupus erythematosus	37 (0.13%)	78 (0.52%)	0.0006
Sjogren syndrome	357 (1.20%)	127 (0.84%)	0.0004
Cataract surgery	16,722 (2.82%)	9707 (3.27%)	0.0009
Co-medication			
Antihistamine	33,739 (10.67%)	53,143 (15.32%)	0.0211
Biguanides	6066 (20.32%)	4705 (31.52%)	0.0588
Dipeptidyl peptidase-4 inhibitor	3254 (10.90%)	4218 (28.26%)	0.3872 *
Sodium-glucose cotransporter-2 inhibitors	497 (1.66%)	550 (3.68%)	0.0785
Beta-blockers	234,147 (39.48%)	154,502 (52.10%)	0.0771
Statin	10,976 (36.77%)	9604 (64.34%)	0.0982
Diuretics	15,079 (50.51%)	10,677 (71.53%)	0.0226
Benzodiazepine	7577 (25.38%)	4646 (31.13%)	0.0168

ASD: absolute standard difference, CHD: coronary heart disease, DM: diabetes mellitus, *N*: number. * Denotes a significant difference between groups.

**Table 2:** The risk of dry eye disease between the two groups.

Event	Mild CHD Group	Severe CHD Group	*p* Value
**DED**			
Person-months	893,417	402,709	
Event	3440	1276	
cHR (95% CI)	Reference	0.819 (0.768–0.874)	
aHR (95% CI)	Reference	0.847 (0.784–1.016)	0.8213
**Severe DED**			
Person-months	979,415	433,199	
Event	37	48	
Crude HR (95% CI)	Reference	2.610 (0.946–7.198)	
aHR (95% CI)	Reference	5.454 (1.551–7.180) *	0.0001 *

aHR: adjusted hazard ratio, CHD: coronary heart disease, cHR: crude hazard ratio, CI: confidence interval, DED: dry eye disease. * Denotes significant difference between groups. Crude HR: The risk of developing DED in severe the CHD group compared to the mild CHD group without controlling for the effect of multiple risk factors including demographic data, co-morbidities, and co-medications. aHR: The risk of developing DED in the severe CHD group compared to the mild CHD group after controlling for the effect of multiple risk factors including demographic data, co-morbidities, and co-medications.

**Table 3:** Subgroup analysis, stratified by sex.

Event	aHR	95% CI	*p* for Interaction
DED			0.5700
Male	0.823	0.768–0.981	
Female	0.871	0.790–1.161	
Severe DED			0.0584
Male	4.917	1.394–7.629	
Female	6.202	2.177–8.227	

aHR: adjusted hazard ratio, CI: confidence interval, DED: dry eye disease.

**Table 4:** Subgroup analysis, stratified by age.

Event	aHR	95% CI	*p* for Interaction
DED			<0.0001 *
<60	0.723	0.640–0.816	
60–69	0.830	0.759–0.997	
>=70	0.908	0.829–1.296	
Severe DED			0.6112
<60	4.968	1.427–9.062	
60–69	5.463	1.826–7.347	
>=70	5.285	1.306–5.398	

aHR: adjusted hazard ratio, CI: confidence interval, DED: dry eye disease. * Denotes a significant difference between the two groups.

## Data Availability

The data used in this study are not available due to the policy of the National Health Insurance Administration of Taiwan.

## Supplementary Materials

The following supporting information can be downloaded at: https://www.mdpi.com/article/10.3390/diagnostics14060586/s1. Table S1: The association between dry eye disease development and potential risk factors.
