# Supplementary material for: The Extents of Coronary Heart Disease and the Severity of Newly Developed Dry Eye Disease: A Nationwide Cohort Study
Source: Diagnostics (Basel). 2024 Mar 10;14(6):586. doi: 10.3390/diagnostics14060586 (PMC10969748; doi:10.3390/diagnostics14060586)
Supplement: Supplementary file 1 [file diagnostics-14-00586-s001.zip › diagnostics-2872790-supplementary.pdf]

diagnostics

MDPI

Table S1. The association between dry eye disease development and potential risk factor.

Factors	aHR	95% CI	P value
Age (Reference:<60)			
60-79	1.489	1.094-1.861	0.0120*
>=80	1.115	0.748-1.379	0.1145
Sex (Reference: Female)			
Male	0.548	0.347-1.160	0.6526
Co-morbidities			
DM	1.216	1.099-1.426	0.0284*
Rheumatic arthritis	1.432	0.875-1.692	0.2263
Systemic lupus erythematous	0.883	0.524-1.097	0.3718
Sjogren syndrome	1.664	1.185-2.372	0.0005*
Cataract surgery	1.280	0.818-1.492	0.4067
Co-medication			
Anti-histamine	1.217	1.051-1.568	0.0314*
Beta-blocker	1.192	0.716-1.777	0.5925
Diuretic	1.504	0.365-1.938	0.5004
Benzodiazepine	1.619	1.004-2.721	0.0401*

aHR: adjusted hazard ratio, CI: confidence interval, DM: diabetes mellitus. * denotes significant correlation to dry eye disease development
